# Real-World Use of Hybrid Closed-Loop Systems during Diabetes Camp: A Preliminary Study for Secure Configuration Strategies in Children and Adolescents

**DOI:** 10.3390/nu16142210

**Published:** 2024-07-10

**Authors:** María José Olid-Cárdenas, Alfonso Lendínez-Jurado, Gabriela Monroy-Rodríguez, Ana Gómez-Perea, Ana Cano-Ortiz, Ana B. Ariza-Jiménez, Ana García-Ruiz, Patricia Jiménez-Cuenca, María José Picón-César, Isabel Leiva-Gea

**Affiliations:** 1 Department of Marketing and Communication, Faculty of Communication, European University of Madrid, 28670 Villaviciosa de Odón, Spain; mj.olid.cardenas@facultyue.es; 2 Faculty of Tourism, University of Malaga, Campus de Teatinos s/n, 29071 Málaga, Spain; 3 Andalucía Tech, Universidad de Málaga, Campus de Teatinos s/n, 29071 Málaga, Spain; isabeleivag@gmail.com; 4 Department of Pediatric Endocrinology, Hospital Regional Universitario de Málaga, 29011 Málaga, Spain; gomezpereana@gmail.com (A.G.-P.); patriciajimenez1795@gmail.com (P.J.-C.); 5 Distrito Sanitario Málaga-Guadalhorce, 29009 Málaga, Spain; anagaruu@gmail.com; 6 Department of Endocrinology and Nutrition, Parc Sanitari Sant Joan de Déu, 08830 Sant Boi de Llobregat, Spain; 7 Instituto de Investigación Sant Joan de Déu, 08950 Barcelona, Spain; 8 Instituto de Investigación Biomédica de Málaga (IBIMA), 29010 Málaga, Spain; mjpiconcesar@gmail.com; 9 Department of Didactics of Experimental, Social and Mathematical Sciences, Faculty of Education, Complutense University of Madrid, 28040 Madrid, Spain; acano07@ucm.es; 10 Department of Pediatric Endocrinology, Hospital Universitario Reina Sofía, 14004 Córdoba, Spain; micodemas@hotmail.com; 11 Faculty of Medicine, University of Córdoba, Av. Menéndez Pidal, 7, 14004 Córdoba, Spain; 12 Instituto Maimónides de Investigación Biomédica de Córdoba (IMIBIC), 14004 Córdoba, Spain; 13 Department of Endocrinology and Nutrition, Hospital Universitario Virgen de la Victoria, 29010 Málaga, Spain; 14 Centro de Investigación Biomédica en Red-Fisiopatología de la Obesidad y Nutrición (CIBEROBN), Instituto de Salud Carlos III, 28029 Madrid, Spain

**Keywords:** advanced hybrid closed loop, diabetes camp, Medtronic MiniMed^TM^ 780G, Tandem Control-IQ, pediatric type 1 diabetes, time in hyperglycemia, time in range

## Abstract

The introduction of closed-loop systems in the pediatric population has been a revolution in the management and evolution of diabetes. However, there are not many published studies in situations in which the feeding, schedules, and activities of the children deviate from the routine for which the systems were programmed, as in the case of a summer camp for children and adolescents with diabetes, where the specific programming of this device is not well known. It was a single-center prospective preliminary study. A total of twenty-seven patients (mean age 11.9 ± 1.9 years, 40% male, duration of diabetes 6.44 ± 2.83 years) were included (twenty with Medtronic MiniMed 780G system and seven with Tandem Control-IQ). Glucometric variables and pump functionality were monitored during the 7-day camp and in the following 3 weeks. There was no decrease from the objective TIR 70% at any moment. The worst results in Time Below Range were at 72 h from starting the camp, and the worst results in Time Above Range were in the first 24 h, with a progressive improvement after that. No episodes of level 3 hypoglycemia or ketoacidosis occurred. The use of specific programming in two integrated systems, with complex blood glucose regulation algorithms and not-prepared-for situations with increased levels of physical activity or abrupt changes in feeding routines, did not result in an increased risk of level 3 hypoglycemia and ketoacidosis for our pediatric type 1 diabetes (T1D) patients, regardless of the closed-loop device.

## 1. Introduction

Hybrid closed-loop systems (HCLSs) aid in the management of hyperglycemia and the reduction in hypoglycemia by automatically adjusting insulin infusion based on real-time continuous glucose sensor readings and predictive algorithms. The application of these systems in children and adolescents diagnosed with type 1 diabetes (T1D) has demonstrated both safety and efficacy, with a high percentage of users achieving glycemic targets [1,2,3,4,5]. In Europe, there are three HCLSs approved for use in the pediatric age group: CamAPS FX (CamDiab, Cambridge, UK), Medtronic MiniMed™ 780G (Medtronic, Northridge, CA, USA), and Tandem Control-IQ AP System (Tandem Diabetes Care, San Diego, CA, USA). The choice of a specific system typically depends on the clinical team’s experience and the child’s age or insulin requirements.

The widespread adoption of HCLSs in children and adolescents presents a challenge for healthcare teams as they learn to adapt system parameters to situations where children’s meals, schedules, and activities deviate from the routine for which the systems were programmed. Diabetes camps exemplify such scenarios. Diabetes camps have been in operation for decades, and their beneficial impact (both metabolic and psychosocial) on children and adolescents with T1D is well established [6,7]. However, reports on the performance and safety of HCLS in diabetes camp settings are still limited. Early exploratory studies have shown that HCLSs can effectively reduce nocturnal hypoglycemia [8,9], but these studies were conducted under highly controlled conditions, making it difficult to extrapolate the findings to real-life scenarios. A preliminary Medtronic HCLS algorithm was tested in a supervised camp setting. The system was configured with an overall glycemic target of 120 mg/dL and 160 mg/dL for exercise mode. The results indicated that the system was safe and achieved a time in range of 70–77% by the end of the week [10]. In another study conducted during a winter camp, the use of Tandem Control-IQ HCLS (Control-IQ) demonstrated improved glycemic control and reduced exposure to hyperglycemia compared to sensor-augmented insulin pumps in children and adolescents [11]. In this study, the Control-IQ system was programmed with the participants’ usual insulin parameters. In a subsequent study that examined the utilization of Control-IQ in adolescents during a 3-day ski camp, it was observed that the system demonstrated both safety and effectiveness. To accommodate the heightened activity levels during the camp, adjustments were made for all participants, including a reduction of 20% in basal and bolus insulin doses, along with the consistent utilization of the exercise mode throughout the day [12]. However, hypoglycemia was still common in all age groups, so prospective studies characterizing individual variables are needed to facilitate tailored insulin dose adjustments that minimize glycemic variability while optimizing control in the diabetes camp setting.

As of the time of writing, no further publications on the performance of HCLSs in diabetes camps were found. Thus, the objective of the present study was to describe the glycemic outcomes of children with type 1 diabetes using two different HCLSs, Medtronic MiniMed 780G and Tandem Control-IQ, during their participation in a diabetes camp. 

## 2. Materials and Methods

This was a single-center prospective preliminary study carried out in a summer camp and the Diabetes Unit of a tertiary hospital in Spain (Regional University Hospital of Malaga), utilizing data extracted from electronic medical records over a one-month follow-up period. Eligible patients were children and adolescents 8 to 15 years of age and a history of type 1 diabetes in treatment with insulin pump therapy, who participated in a 7-day diabetes summer camp. Metabolic variables were monitored at the start of the camp, during the camp, and for 3 weeks thereafter. The main exclusion criteria were a concomitant disease and the use of medications or the presence of other conditions that might influence metabolic control, compromise safety, or prevent participants from completing the study. All participants and their caregivers were informed of the study and signed a consent form. The protocol was approved by the ethics committee of our center.

We designed an action protocol to establish a guide in acute unstable situations such as camps. Sensor alarms for high glucose readings (>180 mg/dL) and low glucose readings (<70 mg/dL) were set with a 20 min prediction time, according to their usual routine. The summer camp monitors recorded all meals and relevant events (e.g., hypoglycemic episodes and exercise) in a diary. These data were also available to the camp physicians so that they could modify each child’s pump settings. Standard treatment procedures and guidelines for diabetes camp were followed. 

All the patients participated in social and physical activities (including swimming pool with its respective disconnections), and they received education in diabetes every day during the camp. Breakfast was served at 9 h, lunch at 14 h, and dinner at 21 h, and patients calculated the amount of rations in order to include them in the pump to cover the meal. A snack was offered at around 12:30 h and 17:30 h. At 00 h, the patients were encouraged to go to bed. Glucose monitoring levels were checked before meals, 2 h after a meal, and at bedtime and then at 3 h. Capillary blood glucose levels were checked if symptoms were discordant with the glucose values obtained by sensor monitoring. Predictive alarms for hypoglycemia were provided by the system’s safety module. 

At the start of the camp, both MiniMed 780G and Control-IQ systems were configured in exercise mode with specific glycemic targets. In this mode, the MiniMed 780G system had a glycemic target of 150 mg/dL, while the Control-IQ system aimed for a glycemic range of 140–160 mg/dL. The children performed this adjustment under the supervision of the camp’s healthcare team, and it had to be activated every 24 h. These targets were set to adapt to the anticipated rise in physical activity during the camp. Furthermore, a 20% increase in grams of carbohydrates per unit of insulin was implemented at dinner with the aim of reducing insulin and being more conservative in preventing hypoglycemia during the night. The duration of insulin action was adjusted to 4 h in MiniMed 780G, and it was increased by 20% of the sensitivity factor of the whole day in Control-IQ.

Metabolic control variables were extracted using the Glooko^®^ (Palo Alto, CA, USA) and CareLink^®^ (Northridge, CA, USA) download platforms at the start of the study and at different cutoff points (24 h, 48 h, 72 h, 7 days, 14 days, 21 days, 1 month). Glycemic targets were defined according to the international consensus on Time In Range (TIR 70–180 mg/dL [3.9–10.0 mmol/L]): >70%; Time Below Range 1 (TBR < 70 mg/dL [<3.9 mmol/L]): <4%; Time Below Range 2 (TBR2 < 54 mg/dL [<3.0 mmol/L]): <1%; Time Above Range 1 (TAR1 > 180 mg/dL [>10.0 mmol/L]) < 25%; and Time Above Range 2 (TAR2 > 250 mg/dL [>13.9 mmol/L]): <5% [13,14]. Achievement of these targets was given in percent. 

Other parameters included were the evaluation of paper charts, total insulin, percentage of insulin in basal or bolus form, the amount of insulin administered as automatic correction, and mean glucose.

Data analysis was performed using free R 4.0.2 software (R-CoreTeam 2020) (https://www.r-project.org/). A Shapiro–Wilk test analysis was performed to determine the normality of the study variables. Results are presented as mean ± SD values in normal distributions or as median (interquartile range [IQR]) in nonnormal distributions. Differences in baseline characteristics, device settings, or glucose measurements between groups, according to the device used, were evaluated using Student *t*-test/Wilcoxon Signed-Rank Test according to the distribution of the data. Linear mixed-effects models were used to determine whether there were differences in glucose measurements over time and between devices. For the estimation of the linear mixed-effects model, a first-order autoregressive matrix (AR1) was chosen due to its better fit according to AIC (Akaike Information Criterion) and BIC (Bayesian Information Criterion). To compare the percentage of children meeting the target at the evaluated moment, the Chi-Squared Test was used. Statistical significance was defined as a *p*-value < 0.05.

## 3. Results

### 3.1. Study Participants

Twenty-seven participants were enrolled (mean age 11.9 ± 1.9 years, 40% male, duration of diabetes 6.44 ± 2.83 years). There were 20 in the MiniMed 780G group and 7 in Control-IQ. Both groups were very homogeneous with few differences, as shown in Table 1.

### 3.2. Glycemic Outcomes

No significant differences were observed in TIR evaluation across the different cut-off points. A pronounced decline in TIR was evident within the first 24 h, reaching its nadir at 14 days (Table 2). Despite this, there was no decline below the target TIR > 70% during the entire follow-up (Figure S1). There were no significant differences in the mean TIR when comparing both systems across the various cut-off points, nor were there significant differences in the percentage of patients achieving a TIR > 70% across the evaluated cut-off points. The points at which more children met targets were at 48 h during the camp and one month after the beginning of the camp (Table 3). 

Significant differences in TBR1 were observed (*p* < 0.02), with the highest percentage of level 1 of hypoglycemia detected at 72 h after the start of the camp and maintained until the end of the camp (Table 2).

No significant differences in TBR2 and TAR1 were observed when evaluating the different cut-off points, nor when comparing both devices in terms of their means or the percentage of patients achieving the target TAR < 25% or TBR2 < 1% (Table 2, Table 3 and Table 4).

Significant differences in TAR2 were detected (*p* < 0.04) across the different cut-off points, with the highest percentage of level 2 hyperglycemia observed from 72 h after the start of the camp and persisting until one week after the camp ended (Table 2). At the start of the camp, statistically significant differences were observed between the two devices with respect to the percentage of patients meeting TAR2 consensus targets < 5% (*p* < 0.021) that were not found at the rest of the follow-up (Table 4).

No significant differences were found when comparing both systems across any of the glucometric variables evaluated at any of the established cut-off points (Table 3).

No significant differences were detected in the percentage of patients achieving target glucometric variables when comparing both systems across the different evaluated cut-off points, except for those observed in TAR2 at baseline (Table 4).

Both systems have demonstrated safety with the established programming, with no detected episodes of level 3 hypoglycemia or ketoacidosis. The comparison of both systems showed no differences in the evaluated points, neither in the comparison of the means of glucometric variables nor in the percentage of patients achieving these glucometric targets. When analyzing both devices globally, significant differences were evident in TBR1 and TAR2 throughout the evaluation.

## 4. Discussion

Diabetes camps for children have traditionally been used as tools for leisure and tourism, with published gains in psychosocial variables for both children and adolescents as well as their primary caregivers. These camps have often been used for evaluating the outcomes of therapy changes, particularly technological implementations [15].

Throughout this year, numerous positions have established integrated systems as the first-line treatment for pediatric type 1 diabetes mellitus. Updated reviews emphasize the need for continued evaluative studies to analyze real-life outcomes and the comparability between devices to better define treatment indications [16,17,18]. This article addresses the need to publish real-life results in acute situations, such as a summer camp, which require adaptive changes in a short period of time. This presents a challenge for initial setup and the evaluation of outcomes when comparing both systems.

Studies utilizing HCLSs in patients’ homes are currently underway, encouraging and enhancing our confidence in these tools. However, there are few reviews on the efforts, challenges, and experiences of using automated insulin delivery systems in environments with high levels of changes that require acute adaptation of HCLSs, such as sports camps, where there is a high level of physical activity and non-usual caregivers [19,20,21].

Studies recruiting children and young people with HCLSs, such as the one by Ng et al. with more than 200 patients, show benefits in glycemic control in variables like TIR and TBR when using HCL for 6 months. This study also evaluates Control-IQ and MiniMed 780G systems and includes results from the camAPS FX system (CamDiab, Cambridge, UK), which was not included in our study [1].

Arrieta et al. stated in a study of 3211 children that more than 75% of users using the MiniMed 780G system achieved glycemic control recommended by the international consensus [4]. This is supported by our study, where in most cut-off points, including the 7-day camp stay and follow-up until one month later, more than 70% of patients achieved the target of TIR > 70%, with up to 80% achieving control in the first 48 h. The nadir (60% of patients achieving the TIR target) occurred at 72 h and another nadir at 14 days post-camp (55%). Understanding the temporal positioning of these nadirs in specific situations could be useful for optimizing adjustments in future camps to achieve optimal glycemic control at these temporal cut-off points.

In the multinational and multicenter DREAM project and other studies conducted in camps, patients treated with an HCLS had less nocturnal hypoglycemia and better glycemic control, with a reduction in TBR and TAR compared to non-automated insulin delivery systems [8,9,19,20,21]. An Italian study published data from a virtual camp with 43 children who recently switched to HCLS, showing that 75% of the children who participated in the camp after starting HCLS could achieve and maintain a TIR of more than 70%. In this case, a TIR of 76% was published with Control-IQ, and after the camp, more than 75% of the participants achieved a TIR of more than 70%. This study presents a different camp model, that being a virtual camp with meals at home. Meals are a challenge in a camp for children with diabetes that could modify glycemic results. Camps with children with type 1 diabetes mellitus share the difficulty of group carbohydrate counting without the primary caregiver’s support, which is a challenge for glycemic control in HCLSs requiring carbohydrate counting for adjustment, as used in our study [22,23].

Studies published on winter sports camps with the Control-IQ system show TIR data throughout the camp duration with a mean of 66.40% [11]. In another 60 h ski camp, with the challenge of sports activity and low temperatures, 20 children showed a trend towards a higher TBR during the camp with the Control-IQ system (median 3.80% vs. 1.40%, *p* = 0.057) [24]. This finding is shared with our study, where we observed a significant increase in TBR1 during and one month after the camp, but without exceeding the recommended TBR < 4% target.

The glycemic results published in our study allow the evaluation of two closed-loop systems (MiniMed 780G vs. Control-IQ) that do not show significant differences in the analyzed glycemic data both during the camp and one month later, using both the means of the glycemic variables and the percentage of patients achieving the consensus targets.

When analyzing the data from both devices globally, two variables show significant differences over the evaluated period: TBR1 and TAR2. Despite the adjustments made to make the algorithm more conservative in both systems, a higher percentage of time in hypoglycemia between 70 and 54 mg/dL (TBR1) was observed, with the highest values at 72 h, but not exceeding the recommended target of TBR < 4% at any cut-off point.

The adjustments made to the systems to avoid hypoglycemia may have impacted the significant increase in TAR2, such as reducing the bolus with a 20% increase in carbohydrate grams per insulin unit at dinner. Additionally, a temporary mode in the Medtronic system suppresses auto-correction boluses, and in the Control-IQ system, there is a 20% increase in insulin sensitivity factor.

This increase in TAR2 during the camp shows its highest figures from 72 h and remains until two weeks after the camp. This may not only be influenced by the conservative settings but also by the challenge of carbohydrate counting in large groups as described.

The established configuration showed safety in acute decompensation variables, as evidenced by the absence of level 3 hypoglycemia or ketoacidosis.

More studies are needed to evaluate glycemic outcomes and specific HCLS configurations to improve knowledge of these algorithms in situations requiring rapid adaptation, such as summer camps with sports activities in pediatric groups with type 1 diabetes mellitus.

## 5. Advantages and Limitations

As a strength, the glycemic evaluation not only during the camp but also three weeks later allows us to assess not only the adaptation of these systems to the acute situation but also the time it takes to recover baseline glycemic results after such an activity and the applied configuration modification.

Among the limitations identified in this study are the small number of participants using the Control-IQ system and the lack of weight data to standardize insulin units in IU/kg/day. In addition, given the small number of total patients included, the data obtained in this study are preliminary and could be expanded with data collected at future camps.

## 6. Conclusions

There are not many studies of integrated systems in special situations in the pediatric population, such as diabetes summer camps. Therefore, the specific programming recommended for such situations is not well known.

This study proposes a configuration of two of the most used systems in the pediatric population, demonstrating safety in acute decompensations, such as level 3 hypoglycemia and ketoacidosis, despite clinical changes along an acute situation with a high level of activity and regardless of the close-loop device. There are no significant differences in glucometric variables when comparing both systems at the different time points evaluated (before camp and three weeks later).

This study enables us to advance in configurability of the closed-loop systems by changing certain parameters that allow the modification of expected results in similar situations, such as summer camps.

## Figures and Tables

**Table 1:** Demographic characteristics and baseline glucose measurements at the beginning of the camp.

	Group N = 27	MiniMed 780G N = 20	Control-IQ N = 7	*p*-Value
Age (y)	11.90 (1.9)	12.20 (1.6)	11 (2.3)	0.162
Male N (%)	11 (40.7)	7 (0.35)	4 (0.57)	0.391
Diabetes duration (y)	6.44 (2.83)	7 (5, 9.75)	3 (2, 4)	0.770
Automatic mode (%)	99 (95, 100)	99.50 (97.2, 100)	92.71 (6.39)	0.026
Glycemia (mg/dL)	143.59 (18.29)	146.40 (19.02)	135.57 (14.22)	0.183
TIR (%)	78.14 (11.20)	76.10 (11.28)	84.00 (9.32)	0.095
TBR1 (%)	1.92 (1.46)	1.95 (1.35)	1.85 (1.86)	0.383
TBR2 (%)	0.22 (0.42)	0.25 (0.44)	0.14 (0.37)	1
TAR1 (%)	15.07 (6.86)	16.15 (6.38)	12.00 (7.76)	0.156
TAR2 (%)	4.55 (5.25)	5.55 (5.74)	1.70 (1.60)	0.385
Total insulin (UI)	35.60 (27.50, 50.20)	39.80 (31.80, 55.22)	32.92 (13.33)	0.385
Basal insulin (%)	39.70 (8.89)	37.20 (7.26)	46.80 (9.75)	0.010
Bolus insulin (%)	60.30 (8.89)	62.80 (7.26)	53.14 (9.75)	0.010
Bolus correction (%)		26.81 (14.45)		
Suspension (%)			3.27 (1.37)	
Morning suspension (%)			15.43 (9.28)	
Afternoon suspension (%)			20.57(6.82)	
Afternoon-Night suspension (%)			27.86 (7.12)	
Night suspension (%)			36 (9.34)	
CHOS (g)	140 (116, 204)	144.70 (41.47)	203.30 (86.29)	0.128

Mean ± SD or median (P25, P75).

**Table 2:** Glucometric results for the entire group during and post-camp (N = 27).

	Arrival	24 h	48 h	72 h	7 d	14 d	21 d	1 m	*p*-Value
Average Glucose (mg/dL)	142.35 (3.42)	145.85 (4.82)	143.58 (3.72)	144.62 (3.48)	148.12 (3.06)	152.85 (3.31)	142.26 (3.19)	138.15 (3.33)	0.03
TIR %	78.14 (11.20)	75.70 (14.83)	76.92 (10.26)	75.81 (9.39)	73.25 (8.29)	72.55 (10.66)	75.81 (12.21)	78.29 (10.11)	0.05
TBR1 %	1.92 (1.46)	2.22 (2.66)	2.62 (2.78)	3.14 (3.00)	2.96 (2.37)	1.70 (1.26)	2.48 (1.86)	2.96 (2.42)	0.02
TBR2 %	0.22 (0.42)	0.51 (1.12)	0.59 (1.62)	0.88 (1.69)	0.59 (0.97)	0.14 (0.36)	0.37 (0.62)	0.40 (0.50)	0.05
TAR1 %	15.07 (6.86)	18.22 (12.13)	15.85 (8.32)	16.33 (7.79)	16.88 (5.50)	19.37 (7.60)	16.48 (7.21)	13.70 (8.17)	0.08
TAR2 %	4.55 (5.25)	3.33 (5.47)	4.00 (4.38)	7.25 (13.09)	6.29 (4.86)	6.18 (5.03)	4.85 (6.71)	3.88 (3.91)	0.04

Data are presented as mean (SD).

**Table 3:** Glucometric differences during and post-camp. Results according to the device.

		Arrival	24 h	48 h	72 h	7 d	14 d	21 d	1 m	*p*-Value
TIR %	MiniMed 780G N = 20	76.10 (11.28)	76.80 (15.73)	77.35 (11.19)	75.3 (9.86)	72.00 (8.68)	71.40 (10.55)	75.60 (11.78)	77.20 (10.43)	0.250
	Control-IQ N = 7	84.00 (9.32)	72.57 (12.39)	75.71 (7.56)	77.28 (8.40)	76.85 (6.20)	75.85 (11.08)	76.42 (14.36)	81.42 (9.10)	
TBR1 %	MiniMed 780G N = 20	1.95 (1.35)	2.20 (2.41)	2.60 (2.56)	3.25 (3.07)	3.10 (2.40)	1.65 (1.13)	2.60 (1.93)	3.15 (2.66)	0.658
	Control-IQ N = 7	1.95 (1.35)	2.20 (2.41)	2.60 (2.56)	3.25 (3.07)	3.10 (2.40)	1.65 (1.13)	2.60 (1.93)	3.15 (2.66)	
TBR2 %	MiniMed 780G N = 20	0.25 (0.44)	0.40 (0.75)	0.50 (1.57)	0.95 (1.66)	0.60 (0.75)	0.15 (0.36)	0.35 (0.58)	0.35 (0.48)	0.757
	Control-IQ N = 7	0.14 (0.37)	0.85 (1.86)	0.85 (1.86)	0.71 (1.88)	0.57 (1.51)	0.14 (0.37)	0.42 (0.78)	0.57 (0.53)	
TAR1 %	MiniMed 780G N = 20	16.15 (6.38)	17.75 (12.94)	15.85 (8.59)	17.25 (8.03)	17.90 (5.47)	20.00 (7.36)	16.70 (6.92)	15.05 (8.10)	0.169
	Control-IQ N = 7	12.00 (7.76)	19.57 (10.22)	15.85 (8.13)	13.71 (6.92)	14.00 (4.83)	17.57 (8.59)	15.85 (8.55)	9.85 (7.64)	
TAR2 %	MiniMed 780G N = 20	5.55 (5.74)	2.85 (5.50)	3.70 (4.68)	7.90 (15.03)	6.40 (5.30)	6.80 (5.41)	4.75 (6.41)	4.25 (4.17)	0.430
	Control-IQ N = 7	1.70 (1.60)	4.71 (5.55)	4.85 (3.57)	5.42 (4.64)	6.00 (3.65)	4.42 (3.50)	5.14 (8.07)	2.85 (3.07)	

Mixed model paired data results. Data are presented as mean (SD).

**Table 4:** Comparison of patients meeting ATTD 2019 consensus targets according to device.

		Arrival	24 h	48 h	72 h	7 d	14 d	21 d	1 m
TIR > 70%	MiniMed 780G (%)	15/20 (75)	16/20 (80)	16/20 (80)	13/20 (65)	14/20 (70)	11/20 (55)	15/20 (75)	17/20 (85)
	Control-IQ (%)	7/7 (100)	5/7 (71.43)	6/7 (85.71)	6/7 (85.71)	7/7 (100)	5/7 (71.43)	5/7 (71.43)	6/7 (85.71)
	*p*-value	0.12	0.33	0.77	0.61	0.33	0.92	0.37	0.96
TBR1 < 4%	MiniMed 780G (%)	18/20 (90)	15/20 (75)	15/20 (75)	12/20 (60)	14/20 (70)	18/20 (90)	20/20 (100)	12/20 (60)
	Control-IQ (%)	7/7 (100)	6/7 (85.71)	6/7 (85.71)	7/7 (100)	6/7 (85.71)	6/7 (85.71)	7/7 (100)	6/7 (85.71)
	*p*-value	0.353	1	1	0.16	0.79	0.76	0.76	0.9
TBR2 < 1%	MiniMed 780G (%)	15/20 (75)	15/20 (75)	16/20 (80)	11/20 (55)	10/20 (50)	17/20 (85)	3/20 (15)	13/20 (65)
	Control-IQ (%)	6/7 (85.71)	6/7 (85.71)	6/7 (85.71)	7/7 (100)	7/7 (100)	7/7 (100)	5/7 (71.43)	4/7 (57.14)
	*p*-value	1	1	0.77	0.11	0.07	0.96	0.54	0.31
TAR1 < 25%	MiniMed 780G (%)	18/20 (90)	17/20 (85)	17/20 (85)	18/20 (90)	18/20 (90)	14/20 (70)	18/20 (90)	18/20 (90)
	Control-IQ (%)	7/7 (100)	5/7 (71.43)	7/7 (100)	7/7 (100)	7/7 (100)	5/7 (71.43)	6/7 (85.71)	7/7 (100)
	*p*-value	0.35	0.19	0.86	0.24	0.35	0.41	0.76	0.39
TAR2 < 5%	MiniMed 780G (%)	11/20 (55)	15/20 (75)	13/20 (65)	11/20 (55)	11/20 (55)	6/20 (30)	15/20 (75)	13/20 (65)
	Control-IQ (%)	7/7 (100)	5/7 (71.43)	4/7 (57.14)	4/7 (57.14)	6/7 (85.71)	4/7 (57.14)	5/7 (71.43)	5/7 (71.43)
	*p*-value	0.021	0.51	0.46	0.81	0.15	0.20	0.65	0.76

## Data Availability

All data are available by contacting the authors.

## Supplementary Materials

The following supporting information can be downloaded at: https://www.mdpi.com/article/10.3390/nu16142210/s1, Figure S1: TIR % for the entire group during and post-camp.
