# Supplementary figures and images for: Real-World Use of Hybrid Closed-Loop Systems during Diabetes Camp: A Preliminary Study for Secure Configuration Strategies in Children and Adolescents
Source: Nutrients. 2024 Jul 10;16(14):2210. doi: 10.3390/nu16142210 (PMC11279836; doi:10.3390/nu16142210)

Figure S1. TIR % for the entire group during and post-camp

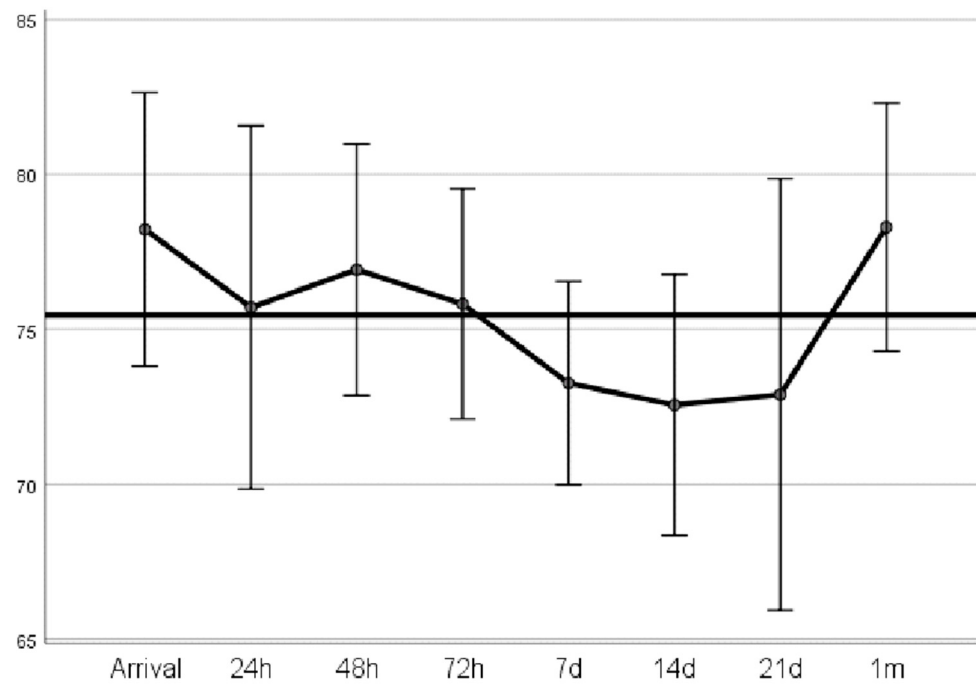

Supplement: Supplementary file 1 [file nutrients-16-02210-s001.zip › nutrients-3083466-supplementary.pdf]
